# Health assessment of French university students and risk factors associated with mental health disorders

**DOI:** 10.1371/journal.pone.0188187

**Published:** 2017-11-27

**Authors:** Antoine Tran, Laurie Tran, Nicolas Geghre, David Darmon, Marion Rampal, Diane Brandone, Jean-Michel Gozzo, Hervé Haas, Karine Rebouillat-Savy, Hervé Caci, Paul Avillach

**Affiliations:** 1 Department of Pediatrics, Hôpitaux pédiatriques de Nice CHU-Lenval, Nice, France; 2 Department of Anesthesiology, Centre Hospitalo-Universitaire Pasteur 2, Nice, France; 3 Department of Preventive Medicine, Université de Nice Sophia-Antipolis, Nice, France; 4 Department of Research in General Medicine, Université de Nice Sophia-Antipolis, Nice, France; 5 Department of Biomedical Informatics, Harvard Medical School, Boston, Massachusetts, United States of America; Medizinische Universitat Wien, AUSTRIA

## Abstract

**Objective:**

The first year of university is a particularly stressful period and can impact academic performance and students’ health. The aim of this study was to evaluate the health and lifestyle of undergraduates and assess risk factors associated with psychiatric symptoms.

**Materials and methods:**

Between September 2012 and June 2013, we included all undergraduate students who underwent compulsory a medical visit at the university medical service in Nice (France) during which they were screened for potential diseases during a diagnostic interview. Data were collected prospectively in the CALCIUM database (*Consultations Assistés par Logiciel pour les Centres Inter-Universitaire de Médecine*) and included information about the students’ lifestyle (living conditions, dietary behavior, physical activity, use of recreational drugs). The prevalence of psychiatric symptoms related to depression, anxiety and panic attacks was assessed and risk factors for these symptoms were analyzed using logistic regression.

**Results:**

A total of 4,184 undergraduates were included. Prevalence for depression, anxiety and panic attacks were 12.6%, 7.6% and 1.0%, respectively. During the 30 days preceding the evaluation, 0.6% of the students regularly drank alcohol, 6.3% were frequent-to-heavy tobacco smokers, and 10.0% smoked marijuana. Dealing with financial difficulties and having learning disabilities were associated with psychiatric symptoms. Students who were dissatisfied with their living conditions and those with poor dietary behavior were at risk of depression. Being a woman and living alone were associated with anxiety. Students who screened positively for any psychiatric disorder assessed were at a higher risk of having another psychiatric disorder concomitantly.

**Conclusion:**

The prevalence of psychiatric disorders in undergraduate students is low but the rate of students at risk of developing chronic disease is far from being negligible. Understanding predictors for these symptoms may improve students’ health by implementing targeted prevention campaigns. Further research in other French universities is necessary to confirm our results.

## Introduction

Students are young adults for whom the first years of university represent a transitory period of vulnerability. The prevalence of depression and anxiety symptoms among students is increasing globally [1–7]. Mental health disorders affect academic performance and physical health, and can lead to suicide [8]. Most psychiatric disorders begin during university years [9], and previous studies have shown higher rates in university students compared to the general population [10].

Several studies have reported that students under stress or with low psychological resilience have a greater tendency to use alcohol to cope with stress [11]. A high prevalence of alcohol drinking in student populations has been reported in 21 European countries [12] as well as worldwide [13]. Heavy drinking is associated with more frequent road traffic accidents, unsafe and unintended sexual activity, personal injury, deaths due to falls, alcohol poisoning and suicide [14]. Furthermore, the use of substances such as tobacco, alcohol and recreational drugs has been well documented among students [13,15,16] and psychiatric disorders are known to exacerbate the negative effects of these substances. Cranford et al. [17] found that cigarette smoking was positively associated with major depression, panic disorder and generalized anxiety disorder. The association between marijuana use and psychiatric disorders is not straightforward: marijuana smokers have shown a consistent positive risk of depression and depressed mood [18] and a reduced level of anxiety [11].

The World Health Organization defines good health as a state of complete physical, mental and social well-being and not merely the absence of disease or infirmity. While students’ health has been assessed in different ways in studies published to date, different scales have been used to assess psychiatric disorders resulting in inconclusive findings. Furthermore, most of the studies were surveys with low-to-moderate participation rates, and either with experienced interviewers filling the forms (i.e., gold standard) or students self-completing standardized questionnaires (i.e., with many expected biases). Overall then, the reported outcomes in most of these surveys were not based on clinical diagnoses made by a physician.

The objective of our study was to assess the mental and physical status and living conditions of undergraduate students enrolled in a French university during an academic year from data collected during a compulsory medical examination by a physician. Risk factors associated with psychiatric symptoms were then estimated.

## Materials and methods

### Study population

This cross-sectional study was conducted between September 2012 and June 2013 among undergraduate university students from 18 faculties (sciences, humanities, medicine and allied programs, law or political science, sports science, engineering and business) of the University of Nice Sophia-Antipolis (UNSA), a large public university in the southeast of France.

We included all undergraduate students who underwent a compulsory medical examination with a physician at the university medical service (UMS) during this period. Students who were reconvened for a control examination or for specific requests such as disability management, dietetic advice, psychological support or drafting of a medical certificate were excluded.

### Procedure

Each year, students are randomly selected by the university to undergo a medical examination to ensure all students are examined during their study cycle. A letter is sent to the home address provided during registration in the APOGÉE database (Application Pour l’Organisation et la Gestion des Enseignements et des Etudiants), a national software program used for university enrollment and administrative file management.

Before the medical examination, a nurse assists students in filling out a questionnaire about their living conditions. The mean duration of the entire visit is 1 hour. The consultations at the UMS of UNSA are provided by one of six medical doctors (D.B., K.R-S., J-M.G., J.S., M.R and N.G.). When necessary, students are referred for a specialized consultation. The National Data Protection Authority approved the study (*Commission Nationale de l’Informatique et des Libertés*, CNIL n° 1421951). As this was an observational study with absence of effect of data collection on patient management and use of anonymized data for the statistical analyses, in accordance with the laws that regulate “non-interventional clinical research” in France (namely articles L.1121-1 and R.1121-2 of the Public Health Code), written informed consent from the participants or the authorization from any other ethics committee to conduct this study was not required.

### Measures

Data were collected prospectively by the medical doctors and nurses using a computer-assisted medical examination software program called CALCIUM (*Consultations Assistés par Logiciel pour les Centres Inter-Universitaire de Médecine*) [19]. This software was created by the University of Lorraine (France) to facilitate the collection of data on students’ health and to provide standard metrics. Each university can edit the portal and collect different data. In the context of this study, forms from the CALCIUM database were anonymized. The questionnaire included information on the students’ demographic characteristics, socioeconomic status, time spent away from studies, career plans or professional objectives, and being informed of future opportunities.

Biometric variables were coded according to current applicable norms; i. the heart rate was considered as abnormal if outside the 60–160 beats per minute range according to the European Resuscitation Council; ii. according to the Join National Committee [20], prehypertension was an observed systolic blood pressure (SBP) between 121 and 139 mmHg or diastolic blood pressure (DBP) between 81 and 89 mmHg, hypertension as an SBP ≥140 mmHg or a DBP ≥90 mmHg, and hypotension as an SBP ≤90 mmHg or DBP ≤60 mmHg; iii. underweight was a body mass index (BMI) ≤18.5, normal weight a BMI between 18.6 and 24.9, overweight a BMI between 25 and 29.9 and obesity a BMI ≥ 30 [21]; iv. near and distance visual acuity was considered as decreased if the score was < 20/20 for both eyes; v. abnormal urinalysis was defined as a positive dipstick test for hematuria (>1+), proteinuria (>1+), leukocyturia (>1+), positive nitrite test or glycosuria. Psychiatric symptoms were defined as students presenting panic attacks, anxiety and/or depressive symptoms based on the clinical experience at the UMS and the Diagnostic and Statistical Manual of Mental Disorders (DSM-IV) criteria. Physicians systematically screened students for: (A) depressive disorder using the following items: depressed mood or irritable, decreased interest or pleasure (anhedonia), change in activity, fatigue or loss of energy; (B) anxiety disorder using the following items: excessive anxiety and worry, restlessness or feeling keyed up or on edge, being easily fatigued, irritability. It is important to note here that the construct of both depression and anxiety is dimensional rather than categorical: the probability of having the disorder is likely to be higher in the group of participants who acknowledge having experienced at least two symptoms. Among the list of symptoms, “anhedonia” and “excessive anxiety and worry” are the core feature of depression and anxiety, respectively. In this way, the size of the groups was sufficient to conduct the planned analysis [22]. If a student answered positively to a simple screening question regarding the occurrence of at least one panic attack in the past year, the clinicians checked the presence of all the DSM-IV symptoms for this disorder. At least four symptoms were needed to qualify for diagnosis. More specifically and according to the DSM-IV classification, the screening for (C) panic attacks was characterized on a limited time by four or more of the following symptoms: (1) palpitations, pounding heart, or accelerated heart rate, (2) sweating, (3) trembling or shaking, (4) sensations of shortness of breath or smothering, (5) feeling of choking, (6) chest pain or discomfort, (7) nausea or abdominal distress, (8) feeling dizzy, unsteady, lightheaded, or faint, (9) feelings of unreality (derealization) or being detached from oneself (depersonalization), (10) fear of losing control or going crazy, (11) fear of dying, (12) numbness or tingling sensations (paresthesia), (13) chills or hot flushes. We defined a positive screening for (A) depressive disorder if the participant presented at least two of the four core symptoms of depression over the past year; (B) anxiety disorder if the participant presented at least two symptoms including “excessive anxiety and worry” over the past year; (C) panic attacks if the participant had experienced at least one episode over the past year.

Alcohol consumption was assessed by the drinking frequency during the preceding year, roughly categorized in two groups [23]: Group 1 encompassing nondrinkers (never-drinker or not during the past year) and occasional drinkers (drinking in the past year but not in the past 30 days or less than 4 days in the past 30 days), and Group 2 encompassing regular and heavy drinkers (drinking ≥ 4 days in the past 30 days). Binge drinking was defined as a positive response to the following question: “over the past two weeks, have you drunk five or more drinks consecutively?” [24]. Tobacco consumption was assessed by the number of cigarettes smoked per day in the previous 30 days and was classified in three levels: Group 1 nonsmokers, Group 2 occasional (≥1 cigarette per day) to regular smokers (between 2 and 10 cigarettes per day), Group 3 frequent smokers (between 11 and 19 cigarettes per day), to heavy smokers (≥20 cigarettes per day). Students who had used recreational drugs during the past 30 days were considered as positive. Poor dietary behavior was defined as an irregular rhythm of meals or unbalanced meals during the past 30 days. The compulsory visits are designed to screen students at risk of unhealthy behavior, risk factors associated with chronic diseases and poor mental health. No data were recorded about suicide risk during the visits. However, as the suicide risk is higher among students with depression and anxiety disorders, alcohol use [25], and obesity [26], any student presenting these conditions were referred for a specialized consultation (information not recorded in the database of the UNSA).

### Statistical analysis

Differences with regards to gender and the field of study were assessed for social and demographic characteristics and clinical diagnosis using a chi-squared test or Fischer’s exact test for qualitative variables. Quantitative variables (i.e. age, blood pressure, heart rate, BMI) were transformed into categorical predictors. If a difference between two variables was statistically significant, we calculated the effect size, which is the magnitude of this difference between groups. An odds ratio (OR) was calculated to measure this effect when a chi-squared test was significant for dichotomous variables. Finally, we fitted three multivariate logistic regression models (dependent variables being the presence/absence of depressive disorder, anxiety disorder and panic attack disorder) to assess the impact of predictors. These were entered in the logistic regression models if they previously showed some statistical significance (p<0.10) in bivariate analyses adjusted for gender and age (S1 Table). Finally, we tested the interactions between psychiatric disorders. The models were fitted by selecting the variables using the Wald test. ORs are expressed with 95% confidence intervals (CI95). The quality of adjustment of the models was tested by the Hosmer-Lemeshow test. The degree of significance was set at p<0.05. We performed chi-squared tests to check whether there were significant differences with regards to the student’s profile (age, gender, year of university, field of study) between those students for whom all data were available and those with any missing data. All statistical analyses were performed using STATA^®^ version 10.0 and R Studio version 3.2.2 for Macintosh^®^.

## Results

### Sociodemographic data and student profiles

**Fig 1:**
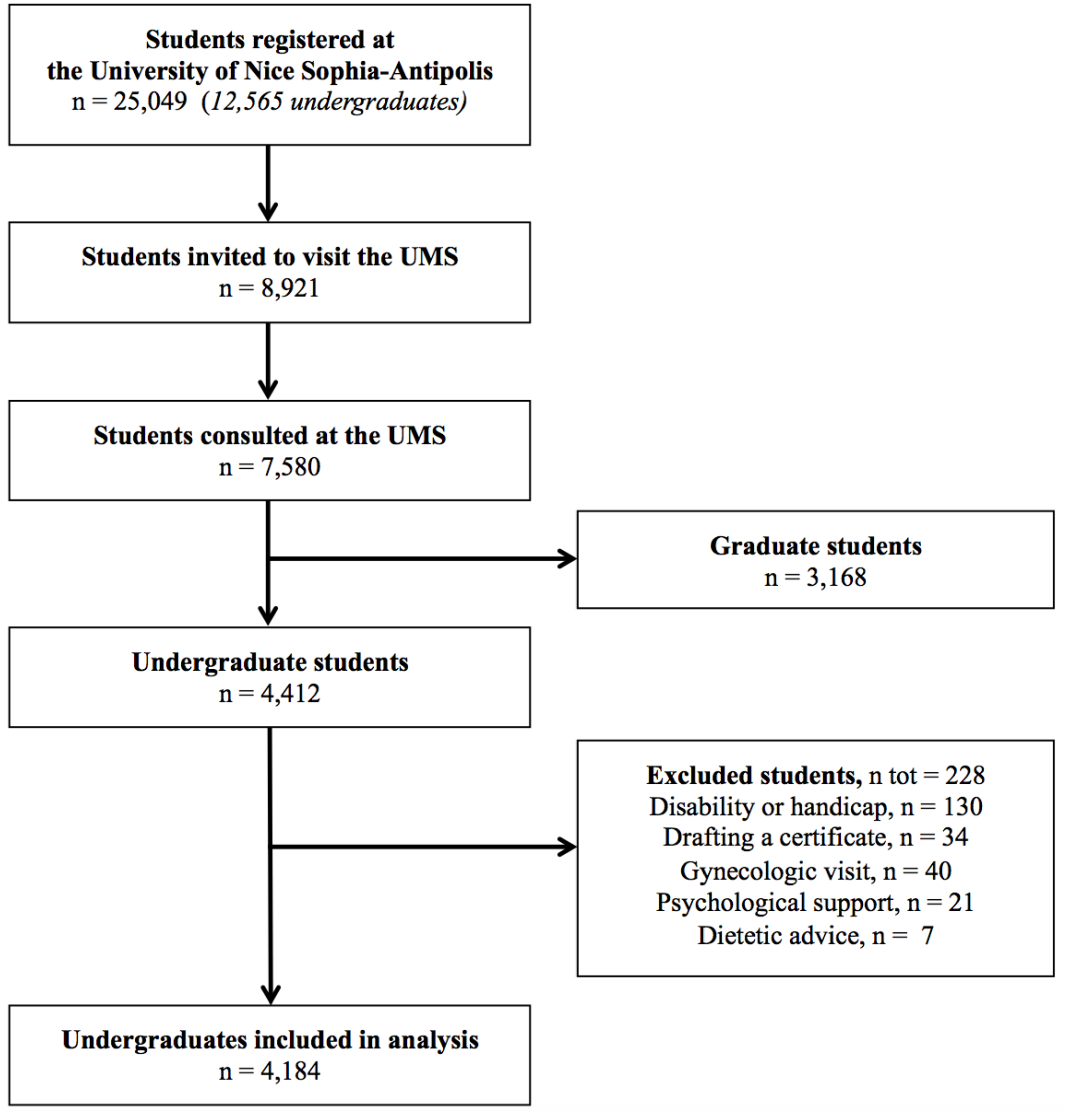
Study flow chart during the academic year 2012–2013.

Overall, 25,049 students were registered in 2012 of whom 12,565 were undergraduates. Among the 8,921 students who were invited to attend the medical examination, 7,580 were examined including 3,168 graduates. Of the 4,412 undergraduates examined, 228 were excluded for various reasons leaving 4,184 students included for final analysis (Fig 1).

First-year students represented 87.86% (CI95 = [86.83; 88.83]) of the undergraduates (Table 1). There were more women (57.43%, CI95 = [55.92; 58.94], p<0.0001) and 35.92% (CI95 = [34.46; 37.40], p<0.0001) were 18 years old. Overall, 27.63% (CI95 = [26.28; 29.01]) of the students studied medicine, 16.66% (CI95 = [15.54; 17.82]) studied the sciences, 14.87% (CI95 = [13.80; 15.98]) studied humanities, 11.74% (CI95 = [10.78; 12.75]) studied law or political science, 5.28% (CI95 = [4.62; 6.00]) studied sports science, and 23.82% (CI95 = [22.54; 25.15]) studied other programs (business, engineering, culture sciences). The proportion of women varied with the field of study, ranging from 18.55% (CI95 = [13.65; 24.31]) in sports science to 72.03% (CI95 = [68.32; 75.52]) in humanities (χ^2^_5_ = 233.05, p<0.0001).

**Table 1:** Student characteristics by gender and by field of study.

	n	All *(n = 4184)*	Men *(n = 1781)*	Women *(n = 2403)*	Sciences *(n = 697)*	Humanities *(n = 622)*	Sports science *(n = 221)*	Law or political science *(n = 491)*	Medicine and allied programs *(n = 1156)*	Other programs^a^ *(n = 997)*
**Age, years**†‡	4184									
<18y		218 (14.69%)	68 (3.80%)	150 (6.24%)	19 (2.73%)	5 (0.80%)	4 (1.81%)	7 (1.43%)	158 (13.67%)	25 (2.51%)
18y		1503 (35.92%)	623 (34.98%)	880 (36.62%)	200 (28.69%)	139 (22.35%)	92 (41.63%)	182 (37.07%)	486 (42.04%)	404 (40.52%)
19y		1151 (27.51%)	483 (27.12%)	668 (27.80%)	197 (28.26%)	182 (29.26%)	71 (32.13%)	153 (31.16%)	283 (24.48%)	265 (26.58%)
≥20y		1312 (31.36%)	607 (34.08%)	705 (29.34%)	281 (40.32%)	296 (47.59%)	54 (24.43%)	149 (30.35%)	229 (19.81%)	303 (30.39%)
**Gender**‡	4184									
man		1781 (42.57%)			362 (51.94%)	174 (27.97%)	180 (81.45%)	181 (36.86%)	441 (38.15%)	443 (44.43%)
woman		2403 (57.43%)			335 (48.06%)	448 (72.03%)	41 (18.55%)	310 (63.14%)	715 (61.85%)	554 (55.57%)
**French nationality**‡	4184	3864 (92.35%)	1644 (92.31%)	2220 (92.38%)	611 (87.66%)	552 (88.75%)	213 (96.38%)	454 (92.46%)	1104 (95.50%)	930 (93.28%)
**Year of university**‡	4184									
first		3676 (87.86%)	1548 (86.92%)	2128 (88.56%)	557 (79.91%)	548 (88.10%)	218 (98.64%)	473 (96.33%)	984 (85.12%)	896 (89.87%)
second		320 (7.65%)	139 (7.80%)	181 (7.53%)	87 (12.48%)	35 (5.63%)	3 (1.36%)	14 (2.85%)	103 (8.91%)	78 (7.82%)
third		188 (4.49%)	94 (5.28%)	94 (3.91%)	53 (7.60%)	39 (6.27%)	0 (0%)	4 (0.81%)	69 (5.97%)	23 (2.31%)
**Learning disabilities**‡	4184	23 (0.55%)	12 (0.67%)	11 (0.46%)	4 (0.57%)	4 (0.64%)	2 (0.90%)	5 (1.02%)	0 (0%)	8 (0.80%)
**Difficulty memorizing lessons**	4184	81 (1.94%)	32 (1.80%)	49 (2.04%)	13 (1.87%)	19 (3.05%)	7 (3.17%)	9 (1.83%)	15 (1.30%)	18 (1.81%)
**Professional objective**‡	4184	2938 (70.22%)	1228 (68.95%)	1710 (71.16%)	423 (60.69%)	462 (74.28%)	173 (78.28%)	338 (68.84%)	817 (70.67%)	725 (72.72%)
**Informed about opportunities**‡	4184	3352 (80.11%)	1425 (80.01%)	1927 (80.19%)	520 (74.61%)	533 (85.69%)	200 (90.50%)	404 (82.28%)	831 (71.89%)	864 (86.66%)

Values are presented as number (percent)

^†^p<0.05 (by gender)

^‡^p<0.05 (by field of studies)

^a^Engineering (schools and institutes), business and economics (schools), arts and culture (schools), higher technician certificate (dietitian institute, social and medical institute)

Most students had professional objectives (70.22%, CI95 = [68.81; 71.60]) with the highest rate in students following the sports science program (78.28%, CI95 = [72.26; 83.53], χ^2^_5_ = 45.58, p<0.0001).

### Living conditions

More women than men reported not living alone (i.e., with a partner and/or child) (OR = 2.18, p = 0.02, CI95 = [1.10; 4.62]), commuting using public transport (χ^2^_2_ = 29.61, p<0.0001), and receiving a scholarship (OR = 1.22, p = 0.003, CI95 = [1.07; 1.40]), while more men than women reported higher levels of physical activity (OR = 1.53, p<0.0001, CI95 = [1.35; 1.74]), living in the parental home (OR = 1.29, p<0.001, CI95 = [1.12; 1.48]) and having public health insurance (OR = 1.25, p<0.001, CI95 = [1.10; 1.42]) (Table 2). Compared with other students, those in medicine were less likely to live with a partner and/or child (p = 0.03), to have only one parent (χ^2^_5_ = 23.99, p<0.001) and at least one parent unemployed (χ^2^_5_ = 25.22, p<0.001), to have a long commute (χ^2^_5_ = 136.96, p<0.0001), deal with financial difficulties (p = 0.002), earn additional income (χ^2^_5_ = 47.50, p<0.0001) and have a lack of regular physical activity (χ^2^_5_ = 321.01, p<0.0001).

**Table 2:** Living conditions, dietary behavior and physical activity by gender and by field of study.

	n	All *(n = 4184)*	Men *(n = 1781)*	Women *(n = 2403)*	Sciences *(n = 697)*	Humanities *(n = 622)*	Sports science *(n = 221)*	Law or political science *(n = 491)*	Medicine and allied programs *(n = 1156)*	Other programs^a^ *(n = 997)*
**Satisfied with living conditions**‡	3670	3483 (94.90%)	1466 (95.13%)	2017 (94.74%)	532 (92.20%)	538 (90.87%)	202 (95.28%)	424 (93.19%)	853 (98.96%)	934 (96.09%)
**Living with a partner/child**†‡	4184	47 (1.12%)	12 (0.67%)	35 (1.46%)	9 (1.29%)	9 (1.45%)	2 (0.90%)	6 (1.22%)	4 (0.35%)	17 (1.71%)
**Parental home**†‡	3470	1954 (56.31%)	876 (59.88%)	1078 (53.71%)	277 (56.42%)	320 (56.34%)	133 (65.84%)	330 (74.16%)	412 (51.05%)	482 (50.37%)
**Having only one parent**‡	4124	1351 (32.76%)	591 (33.60%)	760 (32.14%)	216 (31.44%)	233 (38.39%)	70 (31.96%)	184 (37.94%)	326 (28.65%)	322 (32.59%)
**At least one parent unemployed**‡	4184	209 (5.00%)	95 (5.33%)	114 (4.74%)	28 (4.02%)	49 (7.88%)	11 (4.98%)	36 (7.33%)	38 (3.29%)	47 (4.71%)
**Siblings**‡	4184	3570 (85.33%)	1516 (85.12%)	2054 (85.48%)	577 (82.78%)	514 (82.64%)	200 (90.50%)	411 (83.71%)	1007 (87.11%)	861 (86.36%)
**Long commute**^b^‡	3248	1436 (44.21%)	592 (43.34%)	844 (44.85%)	206 (43.74%)	301 (59.49%)	102 (51.26%)	227 (54.70%)	235 (29.97%)	365 (41.81%)
**Mode of transportation**†‡	3064									
on foot		723 (23.60%)	281 (21.99%)	442 (24.74%)	105 (25.36%)	111 (22.38%)	1 (0.51%)	82 (19.95%)	245 (34.65%)	179 (21.31%)
by public transportation		1597 (52.12%)	623 (48.75%)	974 (54.54%)	228 (55.07%)	306 (61.69%)	84 (42.86%)	214 (52.07%)	319 (45.12%)	446 (53.10%)
by car		744 (24.28%)	374 (29.26%)	370 (20.72%)	81 (19.57%)	79 (15.93%)	111 (56.63%)	115 (27.98%)	143 (20.23%)	215 (25.60%)
**Financial difficulties**‡	4184	26 (0.62%)	16 (0.90%)	10 (0.42%)	5 (0.72%)	8 (1.29%)	2 (0.90%)	5 (1.02%)	0 (0%)	6 (0.60%)
**Grant**†‡	4184	1303 (31.14%)	511 (28.69%)	792 (32.96%)	254 (36.44%)	220 (35.37%)	60 (27.15%)	164 (33.40%)	365 (31.57%)	240 (24.07%)
**Additional income**‡	4184	218 (5.21%)	100 (5.61%)	118 (4.91%)	37 (5.31%)	44 (7.07%)	20 (9.05%)	39 (7.94%)	20 (1.73%)	58 (5.82%)
**Public health insurance**†‡	4184	2483 (59.35%)	1111 (62.38%)	1372 (57.10%)	461 (66.14%)	402 (64.63%)	115 (52.04%)	276 (56.21%)	562 (48.62%)	667 (66.90%)
**Private health insurance**‡	4182	3524 (84.27%)	1519 (85.29%)	2005 (83.51%)	571 (81.92%)	471 (75.72%)	197 (89.14%)	407 (82.89%)	1038 (89.95%)	840 (84.25%)
**C.M.U.**^c^‡	4184	66 (1.58%)	27 (1.52%)	39 (1.62%)	19 (2.73%)	16 (2.57%)	3 (1.36%)	11 (2.24%)	8 (0.69%)	9 (0.90%)
**Bad dietary behavior**	4184									
irregular rhythm of meals‡		1400 (33.46%)	618 (34.70%)	782 (32.54%)	263 (37.73%)	221 (35.53%)	59 (26.70%)	177 (36.05%)	351 (30.36%)	329 (33.00%)
unbalanced meals‡		974 (23.28%)	434 (24.37%)	540 (22.47%)	206 (29.56%)	156 (25.08%)	35 (15.84%)	102 (20.77%)	298 (25.78%)	177 (17.75%)
eating junk food†‡		1873 (44.77%)	862 (48.40%)	1011 (42.07%)	275 (39.45%)	347 (55.79%)	110 (49.77%)	229 (46.64%)	437 (37.80%)	475 (47.64%)
on a diet†‡		70 (1.67%)	17 (0.95%)	53 (2.21%)	15 (2.15%)	12 (1.93%)	4 (1.81%)	7 (1.43%)	13 (1.12%)	19 (1.91%)
irregular rhythm or unbalanced meals‡		1611 (68.50%)	712 (39.98%)	899 (37.41%)	295 (43.32%)	261 (41.96%)	66 (29.86%)	204 (41.55%)	389 (33.65%)	396 (39.72%)
**Physical activity**†‡	4184									
no or occasionally		2516 (60.13%)	966 (54.24%)	1550 (64.50%)	427 (61.26%)	373 (59.97%)	20 (9.05%)	266 (54.18%)	837 (72.40%)	593 (59.48%)
regularly		1668 (39.87%)	815 (45.76%)	853 (35.50%)	270 (38.74%)	249 (40.03%)	201 (90.95%)	225 (45.82%)	319 (27.60%)	404 (40.52%)

Values are presented as number (percent)

^†^p<0.05 (by gender),

^‡^p<0.05 (by field of studies)

^a^Engineering (schools and institutes), business and economics (schools), arts and culture (schools), higher technician certificate (dietitian institute, social and medical institute)

^b^Duration of commute >1 hour per day

^c^“Couverture Medicale Universelle” or Universal Healthcare Coverage: public healthcare insurance for people whose income is lower than a given threshold

The prevalence of irregular rhythm of meals, unbalanced meals and eating junk food were 33.46% (CI95 = [32.03; 34.91]), 23.28% (CI95 = [22.00; 24.59]) and 44.77% (CI95 = [43.25; 46.29]), respectively.

### Physical health

Overweight and obesity affected 18.05% (CI95 = [16.87; 19.28]) of the students included and was more frequent in men than women (OR = 1.50, p<0.0001, CI95 = [1.27; 1.77]) (Table 3). When blood pressure was reported, the prevalence of prehypertension or hypertension overall was 8.34% (CI95 = [7.31; 9.46]) and was more frequently reported in men (OR = 4.24, p<0.0001, CI95 = [3.09; 5.88]). No cases of hypotension or tachycardia were reported. Distance visual acuity was more frequently lower in women (OR = 1.47, p = 0.002, CI95 = [1.15; 1.90]) and more prevalent in students studying medicine and humanities (χ^2^_5_ = 12.86, p = 0.02). Abnormal urinalysis occurred mostly in women (OR = 5.03, p<0.0001, CI95 = [3.14; 8.42]).

**Table 3:** Outcomes of medical examinations, prevalence of psychiatric disorders, and alcohol, cigarette and recreational drug use by gender and by field of study.

	n	All *(n = 4184)*	Men *(n = 1781)*	Women *(n = 2403)*	Sciences *(n = 697)*	Humanities *(n = 622)*	Sports science *(n = 221)*	Law or political science *(n = 491)*	Medicine and allied programs *(n = 1156)*	Other programs^a^ *(n = 997)*
*PHYSICAL EXAMINATION*										
**Overweight and obesity**†‡	3994	721 (18.05%)	365 (21.55%)	356 (15.48%)	144 (22.05%)	114 (19.07%)	34 (15.89%)	109 (23.29%)	154 (14.17%)	166 (17.04%)
**Prehypertension or hypertension**†‡	2650	221 (8.34%)	162 (14.52%)	59 (3.85%)	49 (12.25%)	43 (9.15%)	10 (6.94%)	19 (5.64%)	59 (8.10%)	41 (7.18%)
**Abnormal heart rate**†‡	3798	287 (7.56%)	194 (12.13%)	93 (4.23%)	41 (6.69%)	20 (3.39%)	55 (27.50%)	37 (8.30%)	69 (6.46%)	65 (7.38%)
**Decreased in distant visual acuity**†‡	4184	311 (7.43%)	106 (5.95%)	205 (8.53%)	50 (7.17%)	56 (9.00%)	13 (5.88%)	33 (6.72%)	104 (9.00%)	55 (5.52%)
**Decreased in close visual acuity**	4184	19 (0.45%)	11 (0.62%)	8 (0.33%)	6 (0.86%)	2 (0.32%)	2 (0.90%)	2 (0.41%)	5 (0.43%)	2 (0.20%)
**Abnormal urinalysis**†‡	4184	157 (3.75%)	21 (1.18%)	136 (5.66%)	21 (3.01%)	42 (6.75%)	6 (2.71%)	25 (5.09%)	31 (2.68%)	32 (3.21%)
**Vaccinations up to date**‡	3113	2538 (81.53%)	1100 (81.78%)	1438 (81.33%)	444 (83.30%)	451 (92.23%)	159 (89.83%)	276 (77.75%)	618 (72.79%)	590 (83.10%)
**Control examination needed**	4184	1242 (29.68%)	506 (28.41%)	736 (30.63%)	227 (32.57%)	165 (26.53%)	58 (26.24%)	156 (31.77%)	331 (28.63%)	305 (30.59%)
*PSYCHIATRIC DISORDERS*										
**Anxiety** †‡	4184	317 (7.58%)	82 (4.60%)	235 (9.78%)	53 (7.60%)	61 (9.81%)	4 (1.81%)	47 (9.57%)	90 (7.79%)	62 (6.22%)
**Panic attack** †	4184	43 (1.03%)	8 (0.45%)	35 (1.46%)	9 (1.29%)	4 (0.64%)	0 (0%)	7 (1.43%)	12 (1.04%)	11 (1.10%)
**Depression**	4184	528 (12.62%)	217 (12.18%)	311 (12.94%)	81 (11.62%)	90 (14.47%)	37 (16.74%)	67 (13.65%)	123 (10.64%)	130 (13.04%)
*DRUG USE*										
**Cigarette smoker**‡	3564									
no		2595 (72.81%)	1076 (71.64%)	1519 (73.67%)	407 (76.50%)	393 (69.31%)	158 (75.96%)	321 (72.62%)	698 (79.50%)	618 (65.96%)
occasional or regular		743 (20.85%)	326 (21.70%)	417 (20.22%)	85 (15.98%)	130 (22.93%)	41 (19.71%)	87 (19.68%)	157 (17.88%)	243 (25.93%)
frequent to heavy		226 (6.34%)	100 (6.66%)	126 (6.11%)	40 (7.52%)	44 (7.76%)	9 (4.33%)	34 (7.69%)	23 (2.62%)	76 (8.11%)
**Drinker**†‡	4176									
non- or occasional		4149 (99.35%)	1758 (92.31%)	2391 (97.79%)	689 (98.99%)	622 (100.00%)	219 (99.10%)	489 (99.59%)	1154 (99.83%)	976 (98.59%)
regular to heavy		27 (0.65%)	17 (7.69%)	10 (2.21%)	7 (1.01%)	0 (0%)	2 (0.90%)	2 (0.41%)	2 (0.17%)	14 (1.41%)
**Binge drinking**†‡	4184	190 (4.54%)	137 (7.69%)	53 (2.21%)	29 (4.16%)	22 (3.54%)	17 (7.69%)	14 (2.85%)	34 (2.94%)	74 (7.42%)
**Marijuana use**†‡	3616	362 (10.01%)	225 (14.68%)	137 (6.58%)	65 (12.31%)	74 (13.01%)	21 (10.00%)	41 (9.05%)	52 (5.75%)	109 (11.46%)
**Other recreational drugs**	2911	29 (1.00%)	14 (1.14%)	15 (0.89%)	2 (0.47%)	8 (1.68%)	0 (0%)	2 (0.56%)	5 (0.66%)	12 (1.66%)

Values are presented as number (percent)

^†^p<0.05 (by gender),

^‡^p<0.05 (by field of studies)

^a^Engineering (schools and institutes), business and economics (schools), arts and culture (schools), higher technician certificate (dietitian institute, social and medical institute)

### Psychiatric disorders

The prevalence of depression was 12.62% (CI95 = [11.63; 13.66]) regardless of gender (Table 3). Anxiety affected 7.58% (CI95 = [6.79; 8.42]) of students and was more frequent in women than men (OR = 2.25, p<0.0001, CI95 = [1.72; 2.95]). Students in humanities and in law were significantly the most affected, 9.81% (CI95 = [7.58; 12.42]) and 9.57% (CI95 = [7.12; 12.53]), respectively (χ^2^_5_ = 20.41, p = 0.001).

Panic attacks were less frequently reported than anxiety (1.03%, CI95 = [0.74; 1.38]) and affected more women than men (OR = 3.27, p = 0.002, CI95 = [1.49; 8.20]). In contrast to anxiety, the prevalence of depression and panic attacks did not differ according to the field of study.

The proportion of students who reported high levels of cigarette smoking and alcohol drinking were 6.34% (CI95 = [(5.56; 7.19]) and 0.65% (CI95 = [(0.43; 0.94]), respectively. Binge drinking during the past year was more frequent in men than in women (OR = 3.69, p<0.0001, CI95 = [2.65; 5.21]), particularly in undergraduates studying sports science compared to the others (χ^2^_5_ = 35.89, p<0.0001).

The prevalence of marijuana use and other recreational drug use were 10.01% (CI95 = [9.05; 11.04]) and 1.00% (CI95 = [0.67; 1.43]), respectively, and marijuana was more likely to be consumed by men (OR = 2.44, p<0.0001, CI95 = [1.94; 3.08]), and by students in humanities (χ^2^_5_ = 29.68, p<0.0001).

### Risk factors associated with psychiatric disorders

Difficulties in memorizing lessons and having financial difficulties were positively associated with depression and anxiety (adjusted ORs for difficulties in memorizing lessons: 8.05, CI95 = [4.51; 14.35] and 2.30, CI95 = [1.26; 4.17], respectively; adjusted ORs for financial difficulties: 3.59, CI95 = [1.37; 9.44] and 7.86, CI95 = [3.02; 20.50], respectively). Students with learning disabilities were most at risk of depression and panic attack (ORs = 7.50, CI95 = [2.74; 20.49] and 5.16, CI95 = [1.03; 25.75], respectively) (Table 4).

**Table 4:** Risk factors associated with psychiatric disorders: Depression, anxiety and panic attacks.

		DEPRESSION			ANXIETY			PANIC ATTACKS		
		Adjusted OR [CI95] n = 3670	*p-Wald*	Effect size	Adjusted OR [CI95] n = 3470	*p-Wald*	Effect size	Adjusted OR [CI95] n = 4184	*p-Wald*	Effect size
**Age, years**	<18	0.68 [0.40–1.14]	*0*.*14*		0.63 [0.30–1.31]	*0*.*214*		1.04 [0.23–4.73]	*0*.*964*	
	18	0.91 [0.71–1.17]	*0*.*454*		0.88 [0.63–1.24]	*0*.*463*		0.85 [0.42–1.77]	*0*.*667*	
	19	0.83 [0.63–1.09]	*0*.*187*		1.12 [0.79–1.59]	*0*.*529*		0.72 [0.32–1.63]	*0*.*434*	
	20+	1			1			1		
**Gender**‡§	man	1			1			1		
	woman	0.94 [0.76–1.16]	*0*.*570*		2.28 [1.67–3.11]	*<*.*0001*	2.25 [1.72–2.95]	2.70 [1.22–6.00]	*0*.*015*	3.28 [1.49–8.19]
**Field of study**‡	Sciences				1.48 [0.96–2.30]	*0*.*079*	χ^2^_5_ = 20.41			
	Humanities				1.38 [0.92–2.08]	*0*.*118*				
	Sports science				0.33 [0.11–0.97]	*0*.*044*				
	Law or political science				1.51 [0.97–2.34]	*0*.*069*				
	Medicine and allied programs				1.27 [0.85–1.90]	*0*.*246*				
	Other programs^a^				1					
**Learning disabilities**†§		7.50 [2.74–20.49]	*<*.*0001*	11.04 [4.42–29.03]				5.16 [1.03–25.75]	*0*.*046*	9.57 [1.05–41.26]
**Difficulties in memorizing lessons**†‡		8.05 [4.51–14.35]	*<*.*0001*	13.66 [8.41–22.53]	2.30 [1.26–4.17]	0.006	6.19 [3.67–10.21]			
**Not living in parental home**‡					1.33 [1.02–1.75]	*0*.*038*	1.46 [1.13–1.89]			
**No sibling**‡					1.53 [1.09–2.15]	*0*.*014*	1.33 [0.97–1.81]			
**Dissatisfied with living conditions**†		2.36 [1.63–3.39]	*<*.*0001*	2.98 [2.10–4.18]						
**Financial difficulties**†‡		3.59 [1.37–9.44]	*0*.*010*	13.48 [5.64–34.47]	7.86 [3.02–20.50]	*<*.*0001*	14.84 [6.30–35.41]			
**Anxiety**†§		5.63 [4.22–7.52]	*<*.*0001*	6.56 [5.08–8.44]		*na*		20.26 [8.08–50.84]	*<*.*0001*	13.66 [7.06–24.40]
**Panic attacks**†‡		6.05 [2.66–18.18]	*<*.*0001*	9.11 [4.74–17.72]	12.92 [4.36–38.29]	*<*.*0001*	13.66 [7.06–26.40]		*na*	
**Depression**‡§			*na*		5.52 [4.14–7.36]	*<*.*0001*	6.56 [5.08–8.44]	11.64 [4.81–28.19]	*<*.*0001*	9.11 [4.74–17.72]
**Eating junk food**†		1.30 [1.05–1.60]	*0*.*014*	1.56 [1.30–1.89]						
**Bad dietary behavior**^b^†		1.49 [1.21–1.84]	*<*.*0001*	1.41 [1.17–1.71]						
**Interaction terms**	Anxiety*Panic attacks†		*0*.*032*			*na*			*na*	
	Depression*Panic attacks‡		*na*			*0*.*009*			*na*	
	Depression*Anxiety§		*na*			*na*			*0*.*001*	

Risks are presented as adjusted odds ratios (OR) and 95% confidence intervals (from multivariate analysis models)

Effect sizes are presented as odds ratios (OR) and 95% confidence intervals for binary variables

^†^Predictor significantly associated with depression (p<0.05)

^‡^Predictor significantly associated with anxiety (p<0.05)

^§^Predictor significantly associated with panic attack (p<0.05)

^a^Engineering (schools and institutes), business and economics (schools), arts and culture (schools), higher technician certificate (dietitian institute, social and medical institute)

^b^Irregular rhythm or unbalanced meals

na: not applicable

Students with depressive symptoms were more likely to be dissatisfied with their living conditions (OR = 2.36, CI95 = [1.63; 3.39]) and women were more at risk of anxiety symptoms (OR = 2.28, CI95 = [1.67; 3.11]).

Finally, alcohol drinking, cigarettes smoking and recreational drug use was not related to the psychiatric disorders screened in this study. Comorbidities were found between all psychiatric disorders (p for interaction of the terms significant in the three models): having depression was associated with a 5.6-fold higher risk of having anxiety and a 6.1-fold higher risk of having panic attacks.

## Discussion

This single-center study is one of the largest epidemiological evaluations of undergraduates’ health in France reported to date, including a large sample of university students from 18 faculties. Our results extend the current literature by examining the general health of university students. Their physical and mental health statuses were described using electronic health records collected during compulsory medical visits conducted by six medical doctors, adding to the strength of the findings of the present study.

### Physical health

The prevalence of overweight and obesity in this study was in the mid-range of the prevalence observed in a student population in North America [27] (ranging from 14.8% to 24% depending on the academic discipline) and in Europe [28,29] (ranging from 13.2% to 24.2%). Obesity is associated with an increased risk of comorbidities including cardiovascular disease, diabetes and other metabolic disorders [30]. Furthermore, it has been shown that students are an at-risk group because of a concept known as the “freshman 15”, in reference to the numbers of pounds gained during the first year of university. [31].

Prehypertension and hypertension were less prevalent than in the study reported by Al-Majed et al. [32] where the respective rates were 39.5% and 7%, although blood pressure values were missing for nearly one-third of the students in their study. According to recommendations, hypertension is only diagnosed after repeated measurements. Thus, a single medical visit to the UMS is not adapted for diagnosing hypertension: some students may have had abnormal blood pressure readings because of stress related to the medical visit.

We found that only 3.75% of students had abnormal urinalysis, especially hematuria and leukocyturia, with a significantly higher rate in women. Topham et al. [33] estimated abnormalities in urinalysis in 6.2% of urine samples among university students but only 1% of persistent abnormalities. They concluded that routine screening for abnormalities of urinalysis gives a low diagnostic yield.

We found that 7.43% of students had low visual acuity, with a higher trend for low distance visual acuity. This result might be explained by visual fatigue due to close work [34].

Unlike most published studies to date, our study reports a comprehensive assessment of undergraduates’ physical health. Our data, reflecting an assessment of the first 3 years of university, suggest a fair level of health and we report a similar pattern of values to other published samples. In spite the expected finding of reasonable health, from a public health perspective, the study highlights risk factors for developing certain conditions. In this way, the compulsory and free medical examinations provided by the university help identify students at risk. Students tend not to be overly concerned by their health and may neglect symptoms and postpone a medical consultation until the very last moment. For such students, this medical visit at the UMS may be their first contact with a medical doctor without the presence of their parents. It therefore represents a good opportunity to reinforce prevention information about general health and other specific issues. Screening is of paramount importance to detect, for example, overweight, obesity, and hypertension, as the individual is mostly asymptomatic at early stages of the disease. Furthermore, these are also risk factors for other (cardiovascular, cerebrovascular and renal) diseases. Raising students’ awareness during this medical consultation may change the course of the disease, and hopefully stop it.

### Psychiatric disorders

Findings from the present study indicate that the majority of university students in Nice do not show a higher prevalence of mental disorders than the general population [35]: 12.62% of students reported depressive symptoms, 7.58% reported anxiety symptoms and only 1.03% reported panic attack symptoms. This is much lower than rates found in previous studies. Verger et al. [36] reported 25.7% of psychological distress among first-year students in a university in southeastern France and the prevalence among undergraduates in Europe has been estimated in the range of 19.2% to 40% [4,37–39]. Depressive symptoms were present in 13% and 26% of students in the US and Canada [2,40]. Estimations of the prevalence of anxiety symptoms in students vary somewhat throughout the world: 7.6% in China [41], 13.1% in Iran [42], 21.2% in Iceland [38] and 47.1% in Turkey [10]. We may speculate that these differences are related to the different contexts in the various regions. In China, because of the mandatory “one-child” policy, parents tend to overprotect their child against negative life events [43]. In Iran, the universities of medical sciences which are supervised by the government, have experienced decades of expansion resulting in a phenomenon of over education [44] and Iranian students are faced with the risk of unemployment [45]. There are few data on panic attack symptoms in students. The prevalence of panic attack symptoms has been estimated at 33.1% in the US [46], with 27% of undergraduate students in Turkey reported as having stress symptoms [10] and 55.8% of medical freshers in the UK [37]. Our lower prevalence of psychiatric disorders might be explained by the use of different assessment tools (BDI, DASS, SF36, GHQ, CES-D) and by the different student target samples (freshers or not, medical students or not).

We found that women were more likely to present anxiety symptoms, which is consistent with previous studies [2,10]. No gender effect was found with depressive symptoms similarly to several previous studies [3,5,6,10,37,39,41]. However, some studies have reported higher rates of depressive symptoms among women [2,4,36]. Grant et al. [46] suggest that the social and psychological process could explain gender differences in the emergence of depression during adolescence.

Surprisingly, our study did not show that being a medical student was associated with mental health disorders although psychological stress, burnout, anxiety and depression have frequently been reported frequently among medical students [2,36,37,39,40]. Medical school is renowned as being a stressful environment [47] throughout the cursus. Furthermore, first-year medical students in France are under high pressure compared to other students because of a severe, competitive selection process at the end of the first year to get into year 2 (with only a 10% success rate). However, our results suggest that the stressful environment of medical studies is not sufficient to trigger psychiatric disorders. Other aspects, such as psychological history in the preceding years and living conditions, need to be evaluated.

Unexpectedly, we did not find that professional objectives or being informed of future opportunities had any effect in our study sample. Some studies have reported that students who are satisfied with their education have lower depression [3,10], anxiety and stress scores [2,10] but no previous studies have evaluated students’ career ambitions. In France, after obtaining the bachelor’s degree, most students are not prepared to start a professional career. A significant number of them go on to register in French public universities, with or without grant funding, while waiting for opportunities to arise without needing to think immediately about their future.

Similarly to other studies [3,4,6,7,10,42], we found that students who were not satisfied with their living conditions had a 2.4-fold higher risk of depression and that there was a significant association between financial difficulties and psychiatric disorders.

No association was found between physical inactivity and mental health disorders. Feng et al. [41] reported that physical activity had a protective effect on good quality sleep and improved mental health, but participation in physical activity decreased during the transition from adolescence to early adulthood [48].

Furthermore, we found that students with poor dietary behavior were at a higher risk of depression. No previous studies have evaluated the association between nutrition in students and mental health disorders. However, a poor diet and physical inactivity are known to increase the risk of noncommunicable diseases [49].

Several limitations of the present study deserve to be mentioned. First, the study was cross–sectional and did not allow assessment of the temporal relations between the explanatory and dependent variables. Furthermore, the study sample represented only 33.3% of all undergraduates registered in the university and it cannot be ruled out that undergraduates not participating in this screening were treated in private facilities. This low level is also explained by the difficulty for a single UMS to perform a detailed health check every year for all registered students. The low representation rate of undergraduates from a public university in the southeast of France suggests that our results cannot be generalized to all French university students because of social, demographic and economic differences between the populations of different French regions. Additionally, only undergraduates were screened: our results may be extended for graduates whatever the physical and psychological issues. However, this is a true public health issue that would demand additional resources that are not available today. The question of a repeated evaluation during university years is also relevant as opposed to a single compulsory evaluation. Medical visits at the UMS are compulsory which could bias the selection. Some students may postpone the visit believing their health to be satisfactory, neglecting some symptoms of importance. Others may decide not to honor the visit out of a fear of revealing some health issues. In both cases, the prevalence rates may be biased downward.

Finally, while our study detailed the health status of students, other conditions were not evaluated: past psychological history, level of parental education, satisfaction with education, academic performance and workload. In addition, some variables need to be revised and quantified: e.g., quantity of daily alcohol consumption, use of recreational drugs or duration of physical activity and leisure time per day. However, these accurate assessments would lengthen the duration of the consultation and would require more physician time.

## Conclusion

The level of physical health of undergraduate students in France as assessed at a single medical visit, is fair with a low prevalence of psychiatric disorders. However, risk factors for developing chronic diseases are prevalent. Further research in other universities in France are needed to confirm these results. Nevertheless, compulsory medical visits for students comprise a useful tool to screen students at risk and provide help. This is important as poor health could jeopardize a student’s career success through impaired academic performance.

## Supporting information

S1 Table Risk factors associated with psychiatric disorders: Depression, anxiety, and panic attack. Risks are presented as odds ratios (OR) and 95% confidence intervals
^a^Engineering (schools and institutes), business and economics (schools), arts and culture (schools), higher technician certificate (dietitian institute, social and medical institute)
^b^Irregular rhythm or unbalanced meals
na: not applicable.
(DOC)

S1 Checklist (DOCX)

## Abbreviations

BMI: body mass index (kg/m^2^)
bpm: beats per minute
CI95: 95% confidence interval
CMU: “Couverture Médicale Universelle” (or “Universal Healthcare Coverage”)
DBP: diastolic blood pressure
OR: odds ratio
SBP: systolic blood pressure
UMS: university medical service
UNSA: University of Nice Sophia-Antipolis
