# Supplementary material for: Health assessment of French university students and risk factors associated with mental health disorders
Source: PLoS One. 2017 Nov 27;12(11):e0188187. doi: 10.1371/journal.pone.0188187 (PMC5703533; doi:10.1371/journal.pone.0188187)
Supplement: S1 Table — Risks are presented as odds ratios (OR) and 95% confidence intervals aEngineering (schools and institutes), business and economics (schools), arts and culture (schools), higher technician certificate (dietitian institute, social and medical institute) bIrregular rhythm or unbalanced meals na: not applicable. (DOC) [file pone.0188187.s001.doc]

**S1 Table. Risk factors associated with psychiatric disorders: depression, anxiety and panic** attack

|  |  | **DEPRESSION** | | | | **ANXIETY** | | | | **PANIC ATTACK** | | | |
| --- | --- | --- | --- | --- | --- | --- | --- | --- | --- | --- | --- | --- | --- |
|  |  | Crude OR [CI95] | *p-Wald* | Adjusted OR [CI95] n=3670 | *p-Wald* | Crude OR [CI95] | *p-Wald* | Adjusted OR [CI95] n=3470 | *p-Wald* | Crude OR [CI95] | *p-Wald* | Adjusted OR [CI95] n=4184 | *p-Wald* |
| **Age, years** | <18 | 0.57 [0.34-0.94] | *0.028* | 0.68 [0.40-1.14] | *0.14* | 0.52 [0.27-1.00] | *0.05* | 0.63 [0.30-1.31] | *0.214* | 0.75 [0.17-3.28] | *0.703* | 1.04 [0.23-4.73] | *0.964* |
|  | 18 | 0.90 [0.72-1.12] | *0.342* | 0.91 [0.71-1.17] | *0.454* | 0.78 [0.59-1.03] | *0.081* | 0.88 [0.63-1.24] | *0.463* | 0.82 [0.40-1.66] | *0.574* | 0.85 [0.42-1.77] | *0.667* |
|  | 19 | 0.89 [0.70-1.12] | *0.31* | 0.83 [0.63-1.09] | *0.187* | 0.94 [0.71-1.26] | *0.682* | 1.12 [0.79-1.59] | *0.529* | 0.71 [0.32-1.57] | *0.398* | 0.72 [0.32-1.63] | *0.434* |
|  | 20+ | 1 |  | 1 |  | 1 |  | 1 |  | 1 |  | 1 |  |
| **Gender** | man | 1 |  | 1 |  | 1 |  | 1 |  | 1 |  | 1 |  |
|  | woman | 1.07 [0.89-1.29] | *0.465* | 0.94 [0.76-1.16] | *0.570* | 2.25 [1.73-2.91] | *<.0001* | 2.28 [1.67-3.11] | *<.0001* | 3.28 [1.52-7.08] | *0.003* | 2.70 [1.22-6.00] | *0.015* |
| **Field of study** | Sciences | 0.88 [0.65-1.18] | *0.385* |  |  | 1.24 [0.85-1.82] | *0.265* | 1.48 [0.96-2.30] | *0.079* | 1.17 [0.48-2.85] | *0.725* |  |  |
|  | Humanities | 1.13 [0.85-1.51] | *0.414* |  |  | 1.64 [1.13-2.37] | *0.009* | 1.38 [0.92-2.08] | *0.118* | 0.58 [0.18-1.83] | *0.353* |  |  |
|  | Sports science | 1.34 [0.90-2.00] | *0.149* |  |  | 0.28 [0.10-0.77] | *0.014* | 0.33 [0.11-0.97] | *0.044* | *na* | *na* |  |  |
|  | Law or  political science | 1.05 [0.77-1.45] | *0.746* |  |  | 1.60 [1.08-2.37] | *0.02* | 1.51 [0.97-2.34] | *0.069* | 1.30 [0.50-3.37] | *0.594* |  |  |
|  | Medicine  and allied programs | 0.79 [0.61-1.03] | *0.085* |  |  | 1.27 [0.91-1.78] | *0.158* | 1.27 [0.85-1.90] | *0.246* | 0.94 [0.41-2.14] | *0.883* |  |  |
|  | Other programs^a^ | 1 |  |  |  | 1 |  | 1 |  | 1 |  |  |  |
| **Foreign nationality** |  | 1.38 [1.01-1.89] | *0.043* |  |  | 1.56 [1.08-2.26] | *0.019* |  |  | 1.24 [0.44-3.50] | *0.682* |  |  |
| **Year of university** | first | 1.27 [0.79-2.04] | *0.321* |  |  | 0.92 [0.54-1.56] | *0.759* |  |  | 1.90 [0.26-13.93] | *0.527* |  |  |
|  | second | 0.71 [0.38-1.32] | *0.281* |  |  | 0.38 [0.17-0.84] | *0.017* |  |  | 2.97 [0.34-25.60] | *0.322* |  |  |
|  | third | 1 |  |  |  | 1 |  |  |  | 1 |  |  |  |
| **Learning disabilities** |  | 11.04 [4.75-25.63] | *<.0001* | 7.50 [2.74-20.49] | *<.0001* |  |  |  |  | 9.57 [2.17-42.15] | *0.003* | 5.16 [1.03-25.75] | *0.046* |
| **Difficulties in memorizing lessons** |  | 13.66 [8.59-21.73] | *<.0001* | 8.05 [4.51-14.35] | *<.0001* | 6.19 [3.83-10.02] | *<.0001* | 2.30 [1.26-4.17] | 0.006 | 7.04 [2.70-18.37] | *<.0001* |  |  |
| **Having only one parent** |  | 1.30 [1.07-1.57] | *0.008* |  |  |  |  |  |  |  |  |  |  |
| **Not living in parental home** |  | 1.30 [1.07-1.58] | *0.008* |  |  | 1.46 [1.14-1.87] | *0.003* | 1.33 [1.02-1.75] | *0.038* |  |  |  |  |
| **No sibling** |  |  |  |  |  | 1.33 [0.99-1.80] | *0.059* | 1.53 [1.09-2.15] | *0.014* |  |  |  |  |
| **Unsatisfied with living conditions** |  | 2.98 [2.15-4.14] | *<.0001* | 2.36 [1.63-3.39] | *<.0001* |  |  |  |  |  |  |  |  |
| **Financial difficulties** |  | 13.48 [5.98-30.40] | *<.0001* | 3.59 [1.37-9.44] | *0.010* | 14.84 [6.81-32.38] | *<.0001* | 7.86 [3.02-20.50] | *<.0001* | 13.44 [3.88-46.53] | *<.0001* |  |  |
| **Abnormal heart rate** |  | 1.34 [0.96-1.87] | *0.033* |  |  |  |  |  |  |  |  |  |  |
| **Anxiety** |  | 6.56 [5.13-8.38] | *<.0001* | 5.63 [4.22-7.52] | *<.0001* |  |  |  |  | 13.66 [7.42-25.13] | *<.0001* | 20.26 [8.08-50.84] | *<.0001* |
| **Panic attack** |  | 9.12 [4.96-16.76] | *<.0001* | 6.05 [2.66-18.18] | *<.0001* | 13.66 [7.42-25.13] | *<.0001* | 12.92 [4.36-38.29] | *<.0001* |  |  |  |  |
| **Depression** |  |  |  |  |  | 14.84 [6.81-32.38] | *<.0001* | 5.52 [4.14-7.36] | *<.0001* | 9.12 [4.96-16.76] | *<.0001* | 11.64 [4.81-28.19] | *<.0001* |
| **Cigarette smoker** | no | 1 |  |  |  |  |  |  |  |  |  |  |  |
|  | occasional to regular | 1.21 [0.96-1.53] | *0.104* |  |  |  |  |  |  |  |  |  |  |
|  | frequent to heavy | 1.79 [1.27-2.52] | *0.001* |  |  |  |  |  |  |  |  |  |  |
| **Marijuana user** |  | 1.31 [0.97-1.76] | *0.078* |  |  |  |  |  |  |  |  |  |  |
| **Other recreational drugs** |  | 0.53 [0.12-2.22] | *0.381* |  |  | . |  |  |  |  |  |  |  |
| **Eating junk food** |  | 1.57 [1.31-1.88] | *<.0001* | 1.30 [1.05-1.60] | *0.014* | 1.26 [1.01-1.59] | *0.045* |  |  | 2.10 [1.13-3.91] | *0.019* |  |  |
| **Bad dietary behavior**^b^ |  | 1.41 [1.18-1.70] | *<.0001* | 1.49 [1.21-1.84] | *<.0001* |  |  |  |  | 2.03 [1.11-3.72] | *0.022* |  |  |
| **Interaction terms** | Anxiety*Panic attack |  |  |  | *0.032* |  |  |  | na |  |  |  | na |
|  | Depression*Panic attack |  |  |  | na |  |  |  | *0.009* |  |  |  | na |
|  | Depression*Anxiety |  |  |  | na |  |  |  | na |  |  |  | *0.001* |
