# Supplementary material for: Health assessment of French university students and risk factors associated with mental health disorders
Source: PLoS One. 2017 Nov 27;12(11):e0188187. doi: 10.1371/journal.pone.0188187 (PMC5703533; doi:10.1371/journal.pone.0188187)
Supplement: S1 Checklist — (DOCX) [file pone.0188187.s002.docx]

STROBE Statement—checklist of items that should be included in reports of observational studies

|  | Item No. | Recommendation | Page  No. | Relevant text from manuscript |
| --- | --- | --- | --- | --- |
| **Title and abstract** | 1 | (*a*) Indicate the study’s design with a commonly used term in the title or the abstract | 3 | […]a cross-sectional study […] |
|  |  | (*b*) Provide in the abstract an informative and balanced summary of what was done and what was found | 3 |  |
| Introduction | | | |  |
| Background/rationale | 2 | Explain the scientific background and rationale for the investigation being reported | 4 | […] The prevalence of depression and anxiety symptoms among students is increasing globally.[…]. The World Health Organization has defined good health as a state of complete physical, mental and social well-being and not merely the absence of disease or infirmity […].Students’ health was assessed in different ways in studies published to date. But psychiatric disorders were not assessed with the same scales yielding to equivocal conclusions. And most studies were surveys with low-to-moderate participation rates, and with experienced interviewers filling forms (i.e. gold standard) or students self-completing standardized questionnaires (i.e. with many expected biases). Therefore, outcomes in most of these surveys were not clinical diagnoses made by a physician. |
| Objectives | 3 | State specific objectives, including any prespecified hypotheses | 4 | The objective of our study was to rather assess in a comprehensive way mental, physical and living conditions of undergraduate students enrolled in a French university during an academic year. We followed a clinical approach by collecting data during a mandatory medical examination. Risk factors associated with psychiatric symptoms were then estimated. |
| Methods | | | |  |
| Study design | 4 | Present key elements of study design early in the paper | 5 | This cross-sectional study was […] |
| Setting | 5 | Describe the setting, locations, and relevant dates, including periods of recruitment, exposure, follow-up, and data collection | 5 | […] between September 2012 and June 2013 among university students from 18 faculties (sciences, humanities, medicine and allied programs, law or political science, sports science, engineering and business) of the University of Nice Sophia-Antipolis (UNSA), a large public university in southeast France, ranked as the seventeenth best French university […] |
| Participants | 6 | (*a*) *Cohort study*—Give the eligibility criteria, and the sources and methods of selection of participants. Describe methods of follow-up  *Case-control study*—Give the eligibility criteria, and the sources and methods of case ascertainment and control selection. Give the rationale for the choice of cases and controls  *Cross-sectional study*—Give the eligibility criteria, and the sources and methods of selection of participants | 5 | Subsections: “Study population” & “Procedure” |
|  |  | (*b*) *Cohort study*—For matched studies, give matching criteria and number of exposed and unexposed  *Case-control study*—For matched studies, give matching criteria and the number of controls per case |  | Not applicable |
| Variables | 7 | Clearly define all outcomes, exposures, predictors, potential confounders, and effect modifiers. Give diagnostic criteria, if applicable | 6-8 | Subsection: “Measures” |
| Data sources/ measurement | 8* | For each variable of interest, give sources of data and details of methods of assessment (measurement). Describe comparability of assessment methods if there is more than one group | 6-8 | Subsection: “Measures” |
| Bias | 9 | Describe any efforts to address potential sources of bias | 8 | [… ] adjusted for gender and age […] |
| Study size | 10 | Explain how the study size was arrived at | 5 | Subsection “Procedure” & Fig. 1. Study flow chart […] |

Continued on next page

| Quantitative variables | 11 | Explain how quantitative variables were handled in the analyses. If applicable, describe which groupings were chosen and why | 8 | Quantitative variables (i.e. age, blood pressure, heart rate, BMI) were transformed into categorical predictors […] |
| --- | --- | --- | --- | --- |
| Statistical methods | 12 | (*a*) Describe all statistical methods, including those used to control for confounding | 8 | Differences with regard of gender and the field of study were assessed for social and demographic characteristics and clinical diagnosis using a chi-squared test or Fischer’s exact test for qualitative variables […]  three multivariate logistic regression models to study associations between mental health disorders (dependent variables being the presence/absence of depressive disorder, anxiety disorder and panic attack disorder) to assess the impact of predictor […] they were entered in the logistic regression models providing that they previously showed some statistical significance, p<0.10 in bivariate analysis  […] we tested the interactions between psychiatric disorders  […] using the Wald test […]  Hosmer-Lemeshow test […]  Odds ratios […]The degree of significance was set at p<0.05 |
|  |  | (*b*) Describe any methods used to examine subgroups and interactions | 8 | […] tested the interactions between psychiatric disorders |
|  |  | (*c*) Explain how missing data were addressed | 8 | […] We performed chi-squared test to verify whether there were significant differences with regard the students’ profile (age, gender, year of university, field of study) between those students for whom all data were available and those with any missing data […] |
|  |  | (*d*) *Cohort study*—If applicable, explain how loss to follow-up was addressed  *Case-control study*—If applicable, explain how matching of cases and controls was addressed  *Cross-sectional study*—If applicable, describe analytical methods taking account of sampling strategy |  | Not applicable |
|  |  | (*e*) Describe any sensitivity analyses |  | Not applicable |
| Results | | | | |
| Participants | 13* | (a) Report numbers of individuals at each stage of study—eg numbers potentially eligible, examined for eligibility, confirmed eligible, included in the study, completing follow-up, and analysed | 5  7 | Fig 1. Study flow chart […]  First-year students […] |
|  |  | (b) Give reasons for non-participation at each stage | 5 | Fig 1. Study flow chart […] |
|  |  | (c) Consider use of a flow diagram | 5 | Fig 1. Study flow chart […] |
| Descriptive data | 14* | (a) Give characteristics of study participants (eg demographic, clinical, social) and information on exposures and potential confounders | 8-12 | Table 1,2,3 |
|  |  | (b) Indicate number of participants with missing data for each variable of interest | 8-12 | Table 1,2,3 (counts of participants with missing data are indirectly indicated in the column labelled “n”) |
|  |  | (c) *Cohort study*—Summarise follow-up time (eg, average and total amount) |  | Not applicable |
| Outcome data | 15* | *Cohort study*—Report numbers of outcome events or summary measures over time |  | Not applicable |
|  |  | *Case-control study—*Report numbers in each exposure category, or summary measures of exposure |  | Not applicable |
|  |  | *Cross-sectional study—*Report numbers of outcome events or summary measures | 8,9,10  9,11  9,12  12,13  13,14 | Sociodemographic data and students profiles (Table 1) […].  Living conditions (Table 2) […]  Physical health (Table 3) […]  Psychiatric disorders (Table 3) […]  Risk factors associated with mental health disorders (Table 4) […] |
| Main results | 16 | (*a*) Give unadjusted estimates and, if applicable, confounder-adjusted estimates and their precision (eg, 95% confidence interval). Make clear which confounders were adjusted for and why they were included | 8-10,13  S1 table  8 | In results section, all prevalences cited in the following subsections “Sociodemographic data and students profiles”, “ Living conditions”, “Physical health”,  “Mental health disorders” are reported with 95% confidence interval  In S1_table, odds ratio (OR) are reported as “Crude OR” in bivariate analysis and “Adjusted OR” in multivariate analysis  Table 4 reports only “Adjusted OR”  […]adjusted for gender and age […] |
|  |  | (*b*) Report category boundaries when continuous variables were categorized | 6  6  6  6 | […] the heart rate was considered as abnormal if it was less than 60 beats per minute (bpm) or above 160 bpm […]  […] prehypertension was considered if the observed systolic blood pressure (SBP) was between 121 and 139 mmHg or diastolic blood pressure (DBP) between 81 and 89 mmHg. Hypertension was considered if SBP was equal to or above 140 mmHg or DBP was equal to or above 90 mmHg. Finally, hypotension was observed if SBP was equal to or less than 90 mmHg or DBP was equal to or less than 60 mmHg […]  […] Body mass index (BMI) was categorized as underweight if BMI was equal to or less than 18.5, normal weight if BMI was between 18.6 and 24.9, overweight if BMI was between 25 and 29.9 and obesity if BMI was equal to or above 30 […]  […] Close and distant visual acuity was considered as decreased visual acuity if the score was less than 20/20 for both eyes […] |
|  |  | (*c*) If relevant, consider translating estimates of relative risk into absolute risk for a meaningful time period |  | Not applicable |

Continued on next page

| Other analyses | 17 | Report other analyses done—eg analyses of subgroups and interactions, and sensitivity analyses | 13,14  13  13  13 | Risk factors associated psychiatric disorders […]  Table 4. “depression” (n = 3670 students)  Table 4. “anxiety” (n = 3470 students)  Table 4. “panic attack” (n = 3184 students) |
| --- | --- | --- | --- | --- |
| Discussion | | | | |
| Key results | 18 | Summarise key results with reference to study objectives | 16  16  16  16  17  16  16  16 | In subsection “Physical health”:  […] The prevalence of overweight […] Prehypertension and hypertension were less prevalent  […] We found that only 3.75% of students had abnormal urinalysis  […] We found that 7.43% of students had decreased visual acuity  In subsection “Psychiatric disorders”:  […] 12.62% of students reported depressive symptoms, 7.58% reported anxiety symptoms and only 1.03% reported panic attack symptoms  […] We found that students who were not satisfied with their living conditions had a 2.4-fold higher risk of depression  […] No association was found between physical inactivity and mental health disorders  […] we found that students with bad dietary behaviors had a higher risk of depression |
| Limitations | 19 | Discuss limitations of the study, taking into account sources of potential bias or imprecision. Discuss both direction and magnitude of any potential bias | 18  19  19  19  19  19 | […] First, the study was cross–sectional, it did not allow assessment of the temporal relations between the explanatory and dependent variables  […] proportion of undergraduates represented only 33.3% of all undergraduates registered in the university  […] low representation rate of undergraduates from a public university in the southeast of France suggests that our results cannot be generalized to all French university students  […] only undergraduates were screened  […] the prevalence rates may be biased downward  […] others conditions were not evaluated |
| Interpretation | 20 | Give a cautious overall interpretation of results considering objectives, limitations, multiplicity of analyses, results from similar studies, and other relevant evidence | 19  19  19  19 | […] proportion of undergraduates represented only 33.3% of all undergraduates registered in the university  […] only undergraduates were screened  […]prevalence rates may be biased downward  […] others conditions were not evaluated |
| Generalisability | 21 | Discuss the generalisability (external validity) of the study results | 19  19  19 | […] proportion of undergraduates represented only 33.3% of all undergraduates registered in the university  […] The low representation rate of undergraduates from a public university in the southeast of France suggests that our results cannot be generalized to all French university students  […] only undergraduates were screened |
| Other information | |  | | |
| Funding | 22 | Give the source of funding and the role of the funders for the present study and, if applicable, for the original study on which the present article is based |  | There was no source of funding |

*Give information separately for cases and controls in case-control studies and, if applicable, for exposed and unexposed groups in cohort and cross-sectional studies.

**Note:** An Explanation and Elaboration article discusses each checklist item and gives methodological background and published examples of transparent reporting. The STROBE checklist is best used in conjunction with this article (freely available on the Web sites of PLoS Medicine at http://www.plosmedicine.org/, Annals of Internal Medicine at http://www.annals.org/, and Epidemiology at http://www.epidem.com/). Information on the STROBE Initiative is available at www.strobe-statement.org.
